# Change of Variable-Foreperiod Effects within an Experiment: A Bayesian Modeling Approach

**DOI:** 10.5334/joc.235

**Published:** 2022-07-13

**Authors:** Tianfang Han, Robert W. Proctor

**Affiliations:** 1 Department of Psychological Sciences, Purdue University, West Lafayette, IN, USA

**Keywords:** foreperiod, temporal preparation, non-aging foreperiod distribution, multiple trace theory, Bayesian modeling

## Abstract

The framework of binding and retrieval in action control (BRAC) by Frings et al. (2020) proposed that repetition of any element in the previous trial triggers the retrieval of other elements in the same event file. Consistent with this framework, Los et al. (2014) argued that the temporal relation between the warning signal and the target stimulus on a trial is stored in a distinct memory trace (or, event file). Retrieval of the preceding memory trace, which is triggered by perceiving the same warning signal, leads to sequential foreperiod (SFP) effect. We modeled the data from four experiments using a Bayesian method to investigate whether the SFP effect changes over time. Results of Experiments 1, 3 and 4 support the multiple trace theory of preparation, which predicts an asymmetric sequential foreperiod effect, whereas those of Experiment 2 (extremely short foreperiods) support the repetition priming account by Capizzi et al. (2015). Moreover, the significance of the parameters showed that the asymmetry in Experiments 1 and 3 (non-aging distribution) developed gradually, whereas in Experiment 4 (uniform distribution), this asymmetry was significant from the beginning and did not change over time. Implications of these findings for temporal preparation models and BRAC framework were discussed.

For tasks in which participants make responses to a target stimulus, a neutral warning signal or cue presented before onset of the imperative stimulus can increase the response speed (McCormick et al., 2019; Posner et al., 1973; Woodrow, 1914). Such preparatory activity caused by a preceding uninformative signal or cue is usually referred to as temporal preparation (Los et al., 2014; Müller-Gethmann et al., 2003). The effect of temporal preparation on reaction time (RT) and error percentage (EP) is modulated by the foreperiod, which is the interval between termination of the warning signal and onset of the imperative stimulus (Niemi & Näätänen, 1981). A foreperiod is fixed if it is held constant within a trial block and variable if it randomly changes within a block. Previous studies have shown that for fixed foreperiods, RT shows a “U”-shaped curve: As the foreperiod increases, RT first decreases, reaching its shortest value at about 250-ms foreperiod, and then increases as the foreperiod gets longer (Han & Proctor, 2022a; Müller-Gethmann et al., 2003; see Niemi & Näätänen, 1981, for a review). The fixed-foreperiod effect is thought to be determined by the ease of anticipating onset of the imperative stimulus with the specific interval that is in effect. Some research suggests that the effect of short fixed foreperiods (<300 ms) is the result of phasic arousal (Posner et al., 1973; Tona et al., 2016).

In the variable-foreperiod paradigm, RT is modulated by both the current and the previous foreperiods. When foreperiods are uniformly distributed, plotting RT as a function of the current foreperiod produces a decreasing curve (Los et al., 2001; Steinborn et al., 2008). The slope of the foreperiod-RT curve for the current foreperiod is also modulated by the preceding foreperiod, which is consistent with a role of binding and retrieval (Frings et al., 2020). In a uniform foreperiod distribution where each foreperiod is equally likely to occur, this sequential foreperiod effect (SFP effect) is larger when the current foreperiod is short compared to when it is long (as shown in Figure 1). This asymmetric pattern is typical among studies of the SFP effect (e.g., Los et al., 2001; Steinborn et al., 2009; Steinborn et al., 2010; Vallesi & Shallice, 2007).

**Figure 1:**
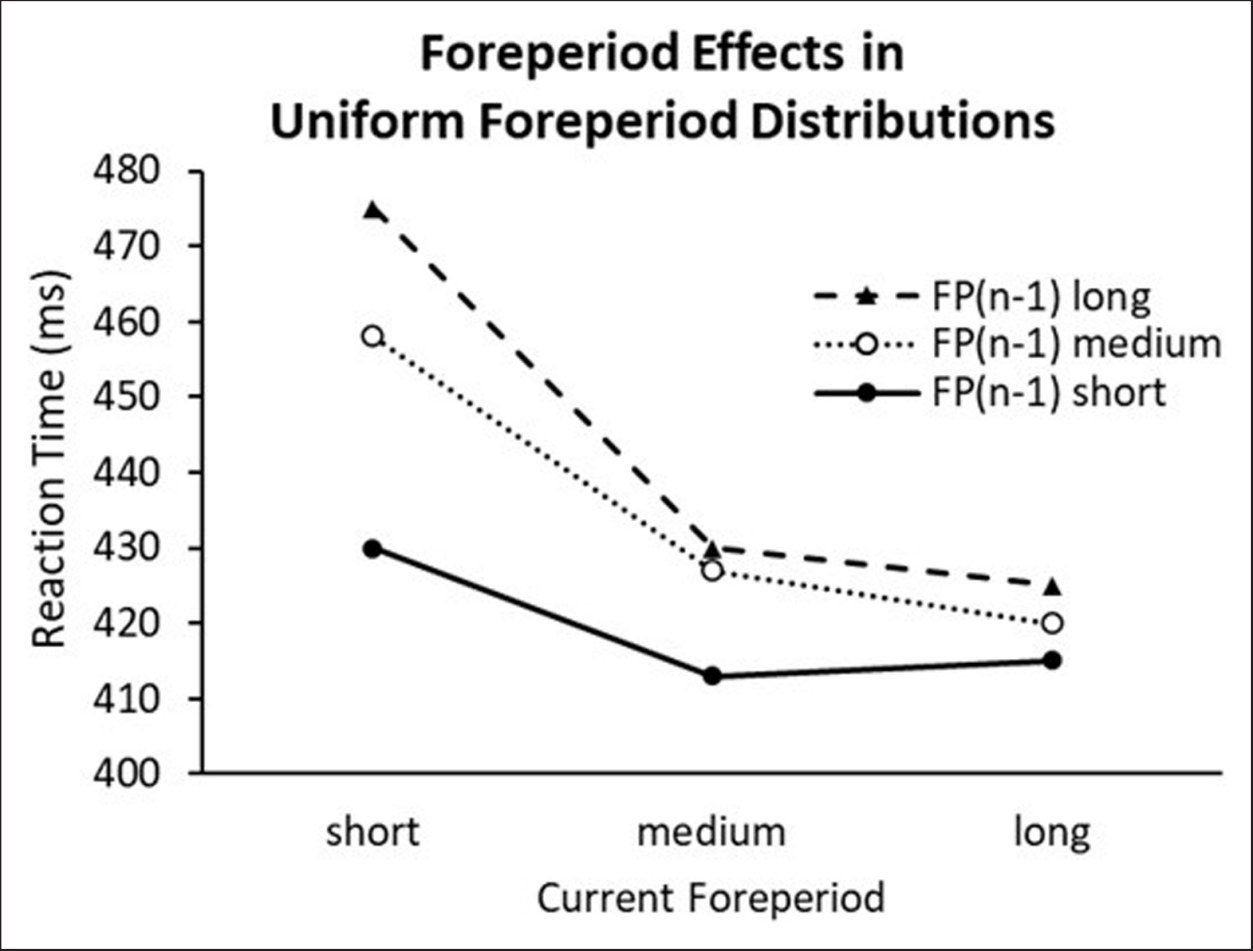
Steinborn et al. (2008): Reaction time as a function of the preceding foreperiod [FP(n-1)] and the current foreperiod in Experiment 1 (long foreperiod set).

## Current Accounts of SFP Effect

In a typical variable-foreperiod task, each possible foreperiod has a *critical moment*, which refers to its expected expiration. Each trial has only one *imperative moment*, which is the same as onset of the imperative stimulus in that trial. The number of critical moments in a trial is determined by the number of distinct foreperiods intermixed within a trial block, and only one critical moment will become the imperative moment for each trial. This relation between the critical moments and the imperative moment has been regarded as an essential tool to explain the mechanism behind the SFP effect.

Niemi and Näätänen (1981) proposed the “expectancy hypothesis”, which states that during the foreperiod, participants develop an expectancy of when the imperative stimulus will appear. The peak of this adaptive expectancy is determined by the conditional probability of the imperative stimulus’s onset (also see Alegria & Delhaye-Rembaux, 1975; Granjon et al., 1973; Stilitz, 1972). In a variable-foreperiod paradigm with a uniform distribution, every time the critical moment of a foreperiod is bypassed, the conditional probability of the imperative stimulus (the hazard function) appearing at later critical moments will increase. After the second longest foreperiod expires, the last critical moment will automatically become the imperative moment, which leads to better preparation because the participant knows with certainty at that point that the imperative stimulus will occur at the longest interval. The hazard function has been used to explain the negative slope of the foreperiod-RT function as well as the decrease of sequential modulation as the foreperiod gets longer. To explain the SFP effect at the shorter foreperiods, it is assumed that participants always expect the current foreperiod to be identical to the preceding one. The limitation of this explanation is that expecting foreperiod repetition is not reasonable when more than two foreperiods are involved.

Dual-process models (Capizzi et al., 2015; Vallesi, 2010) assume that people are able to monitor the hazard function, which leads to the decreasing foreperiod-RT function and the absence of the SFP effect at the longest current foreperiod. Without the influence of the monitoring process, the SFP effect is assumed to be equivalent across different foreperiods. Other than the hazard function, another process is introduced to explain the SFP effect at shorter foreperiods as well as the pattern in cases where endogenous preparation is assumed to be not functioning.

Vallesi and Shallice (2007) found that 4-year-old children showed a symmetric SFP effect, for which a shorter prior foreperiod led to faster current responses on the present trial across all three current foreperiods (1 s, 3 s, 5 s). In contrast, 5- and 6-year-old children showed the typical asymmetric pattern. Vallesi and Shallice concluded that the atypical symmetric SFP effect found in 4-year-old children, whose endogenous processes are not yet developed, is driven by arousal inherited from the prior trial. They assumed that maintaining a high arousal state is effortful. A long foreperiod on the preceding trial costs more energy for maintaining arousal and thus leads to longer RT on the current trial compared to a short preceding foreperiod.

**Figure 2:**
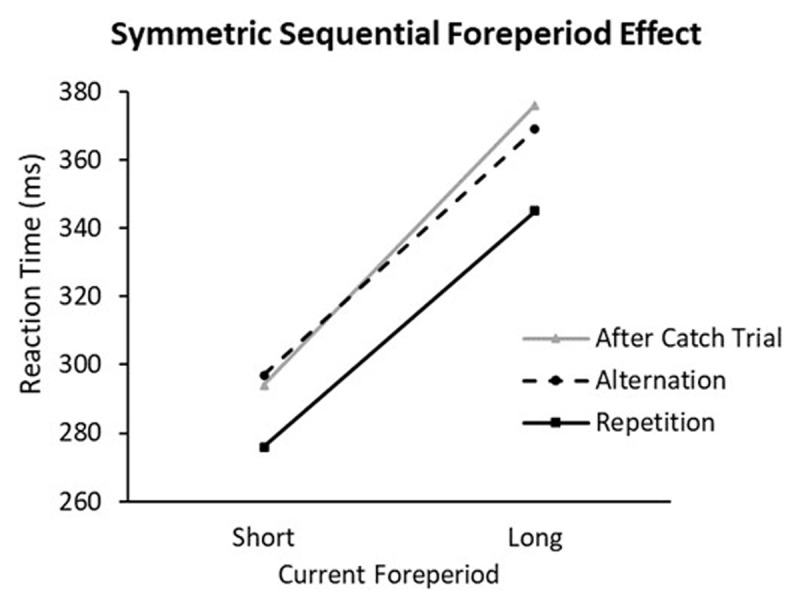
Capizzi et al. (2015): Reaction time as a function of the foreperiod sequence and the current foreperiod in Experiment 2.

The other type of dual process model assumes that the sequential modulation is caused by the memory of the preceding trial. According to the framework of binding and retrieval in action control (BRAC), environmental factors (e.g., warning signal, foreperiod & target stimulus) and cognitive processes that are involved within a trial are integrated into an event-file (Frings et al., 2020). Repetition of any element (warning signal) triggers retrieval of the previous event file. Because in a typical temporal preparation task, the warning signal is kept the same across different foreperiods, this repetition of the warning signal should provide an RT benefit to the repetition of other elements. In other words, foreperiod repetition should lead to faster responses. Following a similar rationale, Yashar and Lamy (2013) and Capizzi et al. (2015) proposed the repetition priming account of SFP effect. Based on this account, when use of the hazard function is discouraged, the SFP effect reflects a repetition benefit of the preceding and the current foreperiods, which should be of similar sizes between short and long foreperiods. This pattern was demonstrated in Capizzi et al. (2015), in which a non-aging foreperiod distribution was used. This special foreperiod distribution contains catch trials and a larger proportion of short foreperiod trials than a uniform distribution so that the hazard function is made flat over time within a trial. Responses were faster when the current and preceding foreperiods were the same and slower when they were different. Moreover, the SFP effects at the shorter and longer foreperiods were estimated to be almost equal in size (as shown in Figure 2).

In contrast with dual-process models, which assume the existence of an endogenous process motivated by monitoring the dynamic change of conditional probability, Los et al. (2014) outlined a multiple trace theory of temporal preparation (MTP). This account links temporal preparation to multiple trace theories of memory, which have been used to explain a variety of memory-related phenomena (e.g., Hintzman, 1986). Similar to the rationale of the BRAC model (Frings et al., 2020), MTP assumes that each previous trial is stored as a memory trace and that the warning signal of the current trial serves as a retrieval cue for all previous memory traces. In addition, MTP makes some specific assumptions regarding the temporal aspect. Within each memory trace, response inhibition is applied to the entire foreperiod duration to avoid premature responding, and activation is only released when approaching the imperative moment. Therefore, in a long-foreperiod memory trace, there is inhibition at the critical moments of short foreperiods, while in a short-foreperiod memory trace, inhibition is not attached to the critical moments of long foreperiods. MTP also assumes that memory becomes more disperse as foreperiod increases and that more adjacent traces produce larger modulation on the current performance. In the current trial, temporal preparation is calculated by aggregating the weighted activation and inhibition values (see Salet et al., 2022, for a computational model of MTP).

With these assumptions, the multiple trace theory is able to predict both a decreasing foreperiod-RT function when the foreperiod distribution is uniform and an asymmetric SFP effect. The SFP effect is regarded as the influence of the preceding memory trace, while the foreperiod-RT function is affected by the whole foreperiod distribution (relative proportions of different foreperiods).

## Short-term or Long-term?

What is shared between MTP and dual-process models is that the SFP effect is a short-term consequence caused by the foreperiod in the preceding trial, which should not be changing over time within an experiment. In other words, this modulation should be similar across trials and blocks. The current study aimed to confirm this characteristic of the SFP effect, as predicted by previous accounts. The majority of previous temporal preparation studies used traditional statistical analyses (e.g., within-subject ANOVAs) on the mean values of different foreperiod conditions, which do not allow further investigation of the influence of previous trials or trial blocks. To avoid this loss of information, we used a Bayesian modeling method as an effective way to extract the trial- and block-level change of the SFP effect. As a first attempt of directly testing whether an effect in a variable-foreperiod paradigm is short- or long-term, the current study was not designed to precisely describe the details of the dynamics or to catch more complex dynamic patterns (e.g., an effect that first increases and then decreases over time). We mainly focused on examining the existence and the general direction of any trial- and block-level changes of the SFP effect. We assumed that this dynamic is a linear function of the previous number of trials and blocks, as a simplified representation of the influence of previous experience.

Part of the data (Experiments 1 and 2) used for modeling in the current study were from Han and Proctor (2022b). Experiment 3 of the current study is a replication of the third experiment in Han and Proctor (2022b), but with more than twice as many participants to increase statistical power. These three experiments were originally designed to test the foreperiod-RT function and the SFP effect with a non-aging foreperiod distribution. Experiment 1 used a long-foreperiod scenario (foreperiods of 400 and 1400 ms), whereas Experiments 2 and 3 used a short-foreperiod scenario (foreperiods of 50 and 200 ms and of 50 and 400 ms, respectively). Experiment 4 is a new one that adopted the same pair of foreperiods as in Experiment 1 but with a uniform distribution, with the aim of investigating the relation between the short- and long-term components of each effect in an aging foreperiod distribution.

In the regression model, we divided the SFP effect into two parameters, one reflecting the influence from arousal and the other representing the priming impact of memory. Based on the dual-process models, the endogenous process is supposed to be discouraged with a non-aging foreperiod distribution. If, in this scenario, the SFP effect is dominated by arousal as argued by Vallesi (2010), only the arousal-related parameter should be significantly different from zero. On the other hand, if the SFP effect follows the repetition priming account of Capizzi et al. (2015), then only the memory-related parameter should be significantly different from zero. If, as stated in the MTP, the SFP effect is the result of retrieving the activation-inhibition rates of the preceding trial, then both parameters should be of similar absolute values, with the memory-related parameter being positive and the arousal-related one being negative, which produces an asymmetric SFP effect. Also, the SFP effect should be immune to the modulation of previous experience, showing no change over time within an experiment. Such a method is informative regarding both confirming the short-term characteristic of the SFP effect and differentiating the predictions from different accounts.

Visalli et al. (2019) and Kruijne et al. (2022) are two rare previous attempts on related topics. The former used a Bayesian way of thinking to describe how participants update the information about the hazard function. A one-trial change of the target stimulus identity was used as a signal, which informed the participants about the change of the hazard function. However, modeling the Bayesian way of thinking is different from the current method, which is using a Bayesian method of parameter estimation to investigate their dynamics within an experiment. In the latter case, a 60-consecutive-trial bundle was used for estimating the parameter values at each time point of the experiment, which was called “rolling regression”. There are several major differences between the current study and Kruijne et al.’s. First, we did not assume that the influence of a trial block is a simple aggregation of the trials it contains. Instead, we regarded them as reflecting different levels of influence from previous experiences. Second, all the experiments of the current study involved catch trials, which were absent in Kruijne et al.’s. In a catch trial, a warning signal is presented but is not followed by an imperative stimulus. The inclusion of catch trials could decrease the equivalence of consecutive-trial bundles. Therefore, the current modeling method is relatively more suitable for our experimental design and research purposes. Moreover, both Visalli et al. and Kruijne et al. focused mainly on the change of the foreperiod-RT function. So far, no studies have explicitly tested the invariance of the SFP effect against the accumulating experience within an experiment.

The current study is also informative for investigating the foreperiod-RT function. Both MTP and the hazard function assume that the foreperiod-RT function is mainly determined by the relative proportions of different foreperiods. Based on both models, this influence is more short-term than long-term since accumulation of previous trials or blocks does not affect the relative foreperiod proportions. Previous research has shown that switching foreperiod distributions can lead to a long-term transfer effect from the prior distribution to the current foreperiod-RT function. Los et al. (2017) showed a within-experiment transfer effect, and Mattiesing et al. (2017) found a transfer effect across two sessions separated by seven days. Within the same foreperiod distribution, the change of foreperiod-RT function has been attributed to a fatigue effect. However, the evidence is mixed, with Bjørklund (1992) showing a larger increase of the RT at the longest foreperiod and Langner et al. (2010) finding no relative RT change between different foreperiods. By assuming the modulation by previous trials and blocks, the current study provided evidence supporting the existence of the fatigue effect only in long foreperiod scenarios.

## Experiments 1, 2, and 3

Experiments 1 and 2 were the first and second experiments in Han and Proctor (2022b). Experiment 3 was a replication (with a larger sample size) of the third experiment. The sample sizes used in the current experiments were larger than those in most temporal preparation research. The main difference between the experiments was the pair of foreperiods involved. The foreperiod distributions in Experiments 1, 2 and 3 were all non-aging.

### Method

#### Participants

A total of 75 (33 male, 42 female, age range: 18–31 years, mean: 19.5 years, SD: 1.9 years), 129 (47 male, 82 female, age range: 18–22 years, mean: 18.5 years, SD: 0.9 years) and 153 (79 male, 74 females, age range: 18–24 years, mean: 19.0 years, SD: 1.2 years) college students participated in Experiments 1, 2 and 3, respectively. All participants in these and the remaining experiment were enrolled in an introductory psychology course at Purdue University and received research credits, reported having normal or corrected-to-normal vision and audition, and were naïve to the purpose of the study. Each participant was only involved in one experiment. All experiments were conducted in accord with a protocol approved by the Purdue University Institutional Review Board and the ethical principles of the American Psychological Association, and all participants signed an approved informed consent form prior to participating.

#### Apparatus and Stimuli

Stimulus presentation and response recording were achieved by means of E-Prime software (Version 2.0, Psychology Software Tools, Inc.) installed on a PC workstation. Participants were seated in front of a 76-cm high table on which an E-Prime response box with a row of five response buttons was placed. Instructions, visual imperative stimulus, and response feedback were presented on a 17-in. LCD monitor in front of the participant, with an unconstrained viewing distance of approximately 63 cm in a dimly lit room. The response box was center-aligned with the display, and participants responded with their left and right index fingers on the leftmost and rightmost buttons of the box.

The imperative stimulus was a lower-case letter (“p” or “q”), which appeared at the center of the display. The size of the letter was 0.5° × 0.3°. The warning signal was a 50-ms, 80-dBA pure tone of 1000 Hz transmitted through a pair of SONY headphones.

#### Procedure

Each trial began with a randomized (uniformly distributed) inter-trial interval ranging between 500 ms and 1500 ms. After the inter-trial interval, the warning tone was presented for 50 ms, after which, for a regular trial, a variable foreperiod started. The foreperiods were 400 ms and 1400 ms, 50 ms and 200 ms, 50 ms and 400 ms in Experiments 1, 2 and 3, respectively. At the expiration of the foreperiod, the imperative stimulus was presented at center of the display. Participants were told to press the left button when “q” appeared and the right button when “p” appeared. The imperative stimulus stayed on the display until a response was made. Error feedback was provided after an incorrect response, while a correct response would directly start the next trial. In a catch trial, the warning tone was followed by a blank slide for 2400 ms, 1200 ms and 1400 ms (one second longer than the long foreperiod) in Experiments 1, 2 and 3, respectively. After the blank slide, a reminder slide saying “No response is needed” was presented for 1500 ms before the next trial began.

Each participant went through one practice block followed by 15 test blocks in Experiments 1 and 2. There were 17 test blocks in Experiment 3. The practice block contained 16 trials – 8 with the shorter foreperiod, 4 with the longer foreperiod, and 4 catch trials – to provide a general impression about the mapping and the structure of a block. Each test block contained 32 trials – 16 with the shorter foreperiod, 8 with the longer foreperiod, and 8 catch trials. Trials with different foreperiods and catch trials were randomly mixed in each block.

Before the experiment, participants were told the mapping they were to use and to maintain their index fingers on the corresponding keys for responding. Speed and accuracy of responses were equally emphasized. Mapping information was included in an introductory slide at the beginning of each block. The experimenter stayed in the room with the participant for all the trials in Experiment 1 but not in Experiments 2 and 3 to obey the social distancing guidance of the Covid-19 protocol.

#### Bayesian Modeling

The modeling process in these and the other experiments was conducted using the *map2stan* function in the *rethinking* R package (R version: 4.1.0; package version 2.13). There were several assumptions underlying the model: First, RT at any trial in any block was assumed to be the sum of a baseline RT and the modulation by the variable-foreperiod effects (including the effect of Current Foreperiod and the SFP effect, which is divided into arousal-based and priming-based components):

RT_trial t+1, block b+1_ = β_baseline_ + β_current foreperiod_ + (β_SFP_arousal_ + β_SFP_priming_)

All the parameters (β) were assumed to be changing over time during an experiment. By using *t* to denote the number of previous trials within a trial block, and *b* to denote the number of previous blocks, each parameter β at trial *t+1* in block *b+1* was assumed to be a linear function of *t* and *b*:

β = α_0_ + α_trial_ × t + α_block_ × b

The intercept of this linear function represents the short-term component of this parameter, which is assumed to reflect the part not modulated by previous experience. The effects of the long-term components are represented by the sum of two products, one of *t* and a trial-related slope and the other of *b* and a block-related slope. We arbitrarily set the RT at the shorter foreperiod under foreperiod repetition as the baseline RT. And the intercept of this baseline parameter was assumed to be vary among participants while all of the other parameters were not. Relatively broad and uninformative prior distributions were used to estimate the values of all parameters.

In the model above, the SFP effect is divided into arousal- and priming-based components. The arousal-based component is assumed to reflect the change caused by different previous foreperiod conditions (short vs. long) while the priming-based component is assumed to represent the change caused by different foreperiod sequence conditions (repetition vs. alternation). The advantage of this division can be explained in this way. In Experiments 1, 2 and 3, a non-aging foreperiod distribution was used with different foreperiod pairs. Based on the dual-process models, the hazard function should have little influence on the SFP effect. If the priming-based component is significantly different from zero while the arousal-based component is not, then the results support a symmetric SFP effect as shown in Figure 2. If it is the opposite case, then the results support a symmetric SFP effect as shown in Vallesi and Shallice (2007). If, however, both parameters were found to be significantly different from zero, then the results favor an asymmetric SFP effect predicted by MTP. Detailed information and original code can be found online.

For each experiment, we constructed five models: full model (Model1), model excluding the short-term component of β_SFP_arousal_ (Model1’),^1^ model excluding trial-related terms (Model2), model excluding block-related terms (Model3), and model excluding both trial- and block-related terms (Model4). After the modeling process, we first compared the performance of the five models, picked the best one and then used it for testing the values of the parameters. An HDI (highest density interval) + ROPE (region of practical equivalence) test was adopted for judging whether a parameter’s value was significantly different from zero (Kruschke, 2018). For each parameter, we calculated the 95% HDI, and computed the ROPE to zero by the effect size at half of Cohen’s conventional definition of a small effect (δ = ±0.1), both of which are rules of thumb suggested by Kruschke. A parameter’s value was judged as not being zero if its HDI fell completely outside the ROPE.

### Results

All trials with RT < 100 ms or >1000 ms were regarded as outliers and excluded from the analyses. To precisely measure the SFP effects, catch trials, the first trial of each block, trials following an incorrect response or a catch trial were also discarded. The proportion of excluded regular trials (non-catch trials) are 31.25%, 31.45% and 32.73% for Experiments 1, 2 and 3, respectively.

#### Experiment 1

Model comparison results (using the *compare* function in the *rethinking* R package) indicate that *Model1’* had the best performance in predicting future data. The 95% interval of the WAIC (widely applicable information criterion) difference between *Model1’* and the second best one (Model1) is [1.17, 4.63], supporting the results based on Akaike weights (0.8 for *Model1’*, 0.18 for *Model1* and 0.02 for the others). The Akaike weight estimates the probability, relative to the other considered models, that a given model is a valid model. It simultaneously considers the model fit to the data and the redundant model flexibility that would allow it to fit noise. Given its good balance of these terms, we used *Model1’* for further testing.

**Figure 3:**
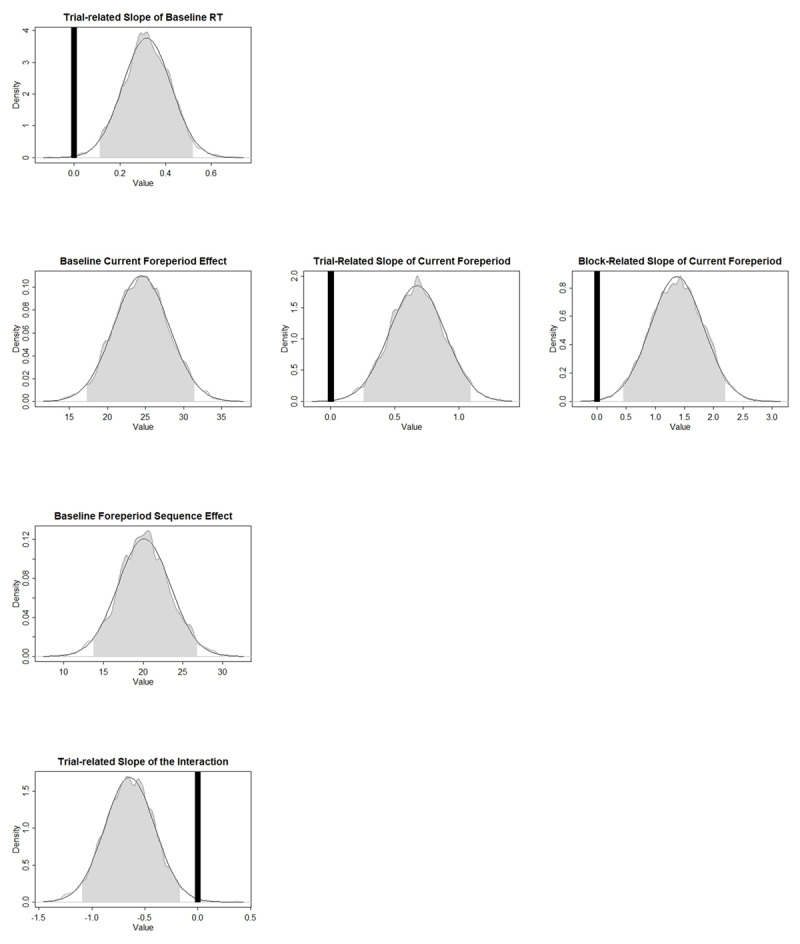
Experiment 1: ROPEs and HDIs of the critical parameters. Gray shades represent the 95% HDIs. Black areas represent the ROPEs to zero. Foreperiod Sequence effect refers to the priming component and the Interaction refers to the arousal component of the sequential foreperiod effect.

**Figure 4:**
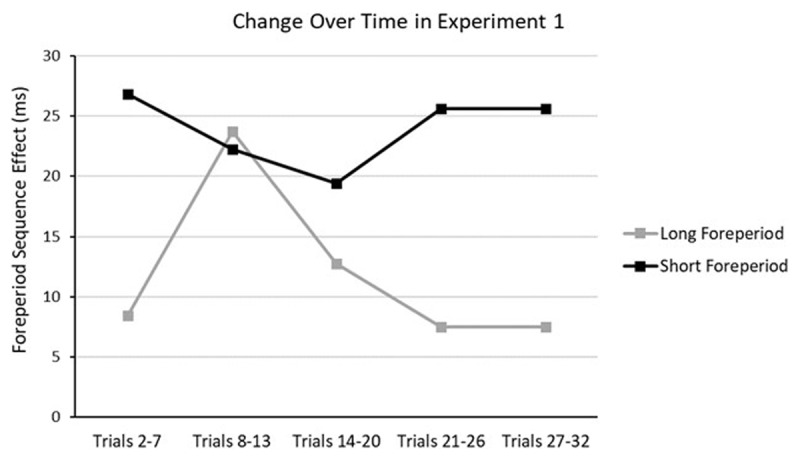
Experiment 1: Foreperiod sequence effect (in ms) as a function of trial bin and current foreperiod. Positive values represent shorter RTs with foreperiod repetition. Two participants were excluded because of missing data points for one or more foreperiod conditions.

Table 1 shows the parameter characteristics of *Model1’*, and Figure 3 presents the ROPEs and HDIs of the parameters that were found to be significantly different from zero. More specifically, the baseline RT was found to increase over trials within a block. The trial- and block-related slopes of the Current Foreperiod effect have positive values significantly different from zero, indicating that the foreperiod-RT function was increasing at the beginning and then became more increasing across trials and blocks. The priming component of the SFP effect shows a repetition benefit at baseline and did not change over time. Surprisingly, in *Model1’*, trial-related slope of the arousal-based component was significantly different from zero. In terms of the data pattern, it means that the benefit of foreperiod repetition at the longer foreperiod decreases over time within a block. Figure 4 roughly demonstrates how the effect sizes at different foreperiods changed across trials.

**Table 1:** Posterior characteristics of the parameters in Experiment 1. CF refers to the effect of the current foreperiod (positive values indicate that RT was shorter at the short foreperiod). For the sequential foreperiod effect, FS effect refers to the priming component (positive values indicate that RT was shorter with foreperiod repetition), and the IN refers to the arousal component (negative values mean that RT was shorter when the foreperiod of the preceding trial was short).


PARAMETER	MEAN	SD	ROPE TO ZERO	95% HDI	REJECT ZERO?

Baseline_trial_	0.32	0.11	[–0.011, 0.011]	[0.11, 0.52]	Yes

Baseline_block_	–0.40	0.22	[–0.022, 0.022]	[–0.83, 0.02]	No

CF	24.45	3.62	[–0.362, 0.362]	[17.30, 31.53]	Yes

CF_trial_	0.67	0.22	[–0.022, 0.022]	[0.24, 1.09]	Yes

CF_block_	1.37	0.45	[–0.045, 0.045]	[0.48, 2.25]	Yes

FS	20.14	3.31	[–0.331, 0.331]	[13.57, 26.68]	Yes

FS_trial_	0.33	0.16	[–0.016, 0.016]	[0.01, 0.65]	No

FS_block_	–0.24	0.34	[–0.034, 0.034]	[–0.92, 0.41]	No

IN	–	–	–	–	–

IN_trial_	–0.64	0.24	[–0.024, 0.024]	[–1.11, –0.18]	Yes

IN_block_	–0.76	0.52	[–0.052, 0.052]	[–1.79, 0.25]	No


#### Experiments 2 and 3

For Experiment 2, model comparison results indicate that *Model1’* has the best performance in predicting future data. The 95% interval of the WAIC difference between *Model1’* and the second best one (Model1) is [1.06, 6.14], supporting the results based on Akaike weights (0.86 for *Model1’*, 0.14 for *Model1* and 0 for the others). Therefore, we used *Model1’* for further testing of the parameters. With regard to Experiment 3, model comparison results indicate that *Model1’* has the best performance in predicting future data. The 95% interval of the WAIC difference between *Model1’* and the second best one (Model1) is [–0.52, 3.72], indicating that the performance of the two models did not differ much from each other, which is consistent with the smaller difference in Akaike weights (0.69 for *Model1’*, 0.31 for *Model1* and 0 for the others). Since limited evidence is still favoring *Model1’*, we decided to use this model for further testing.

**Figure 5:**
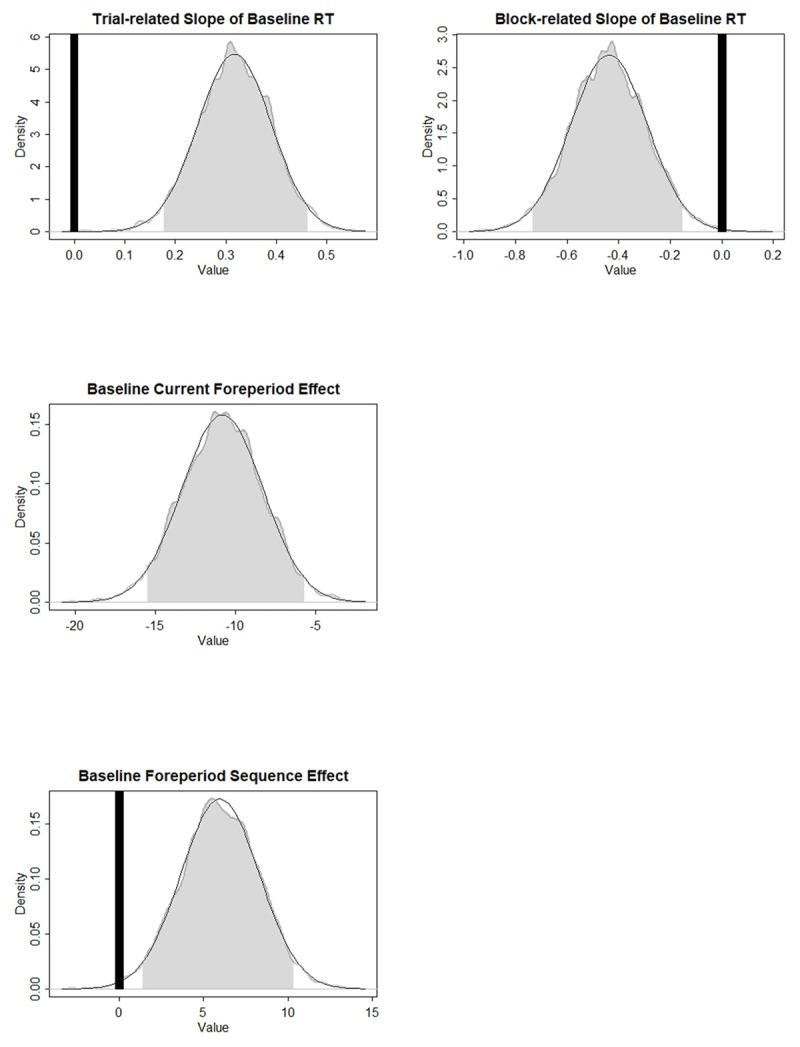
Experiment 2: ROPEs and HDIs of the critical parameters. Gray shades represent the 95% HDIs. Black areas represent the ROPEs to zero. Foreperiod Sequence effect refers to the priming component and the Interaction refers to the arousal component of the sequential foreperiod effect.

Tables 2 and 3 show the parameter characteristics of *Model1’* for Experiments 2 and 3, respectively. Figure 5 presents the ROPEs and HDIs of the parameters that were found to be significantly different from zero in Experiment 2. More specifically, the baseline RT increased over trials within a block, but decreased as the number of previous blocks increased. Only the short-term Current Foreperiod effect and the short-term priming-based SFP effect were significantly different from zero, with the values supporting a decreasing foreperiod-RT function and benefit of foreperiod repetition. Combining the model comparison and the parameter results, the results from Experiment 2 showed no evidence of the arousal component’s existence.

**Table 2:** Posterior characteristics of the parameters in Experiment 2. CF refers to the effect of the current foreperiod (positive values indicate that RT was shorter at the short foreperiod). For the sequential foreperiod effect, FS effect refers to the priming component (positive values indicate that RT was shorter with foreperiod repetition), and the IN refers to the arousal component (negative values mean that RT was shorter when the foreperiod of the preceding trial was short).


PARAMETER	MEAN	SD	ROPE TO ZERO	95% HDI	REJECT ZERO?

Baseline_trial_	0.32	0.07	[–0.007, 0.007]	[0.18, 0.46]	Yes

Baseline_block_	–0.44	0.15	[–0.015, 0.015]	[–0.73, –0.15]	Yes

CF	–10.82	2.53	[–0.253, 0.253]	[–15.79, –5.88]	Yes

CF_trial_	–0.09	0.15	[–0.015, 0.015]	[–0.37, 0.20]	No

CF_block_	0.42	0.32	[–0.032, 0.032]	[–0.20, 1.04]	No

FS	5.98	2.31	[–0.231, 0.231]	[1.43, 10.46]	Yes

FS_trial_	0.01	0.11	[–0.011, 0.011]	[–0.21, 0.23]	No

FS_block_	–0.36	0.24	[–0.024, 0.024]	[–0.82, 0.09]	No

IN	–	–	–	–	–

IN_trial_	–0.06	0.16	[–0.016, 0.016]	[–0.38, 0.26]	No

IN_block_	–0.35	0.36	[–0.036, 0.036]	[–1.05, 0.34]	No


**Table 3:** Posterior characteristics of the parameters in Experiment 3. CF refers to the effect of the current foreperiod (positive values indicate that RT was shorter at the short foreperiod). For the sequential foreperiod effect, FS effect refers to the priming component (positive values indicate that RT was shorter with foreperiod repetition), and the IN refers to the arousal component (negative values mean that RT was shorter when the foreperiod of the preceding trial was short).


PARAMETER	MEAN	SD	ROPE TO ZERO	95% HDI	REJECT ZERO?

Baseline_trial_	0.50	0.06	[–0.006, 0.006]	[0.38, 0.64]	Yes

Baseline_block_	0.81	0.11	[–0.011, 0.011]	[0.58, 1.02]	Yes

CF	1.68	2.22	[–0.222, 0.222]	[–2.57, 6.02]	No

CF_trial_	–0.04	0.13	[–0.013, 0.013]	[–0.29, 0.22]	No

CF_block_	0.45	0.24	[–0.024, 0.024]	[–0.02, 0.92]	No

FS	13.68	2.05	[–0.205, 0.205]	[9.57, 17.72]	Yes

FS_trial_	–0.04	0.10	[–0.010, 0.010]	[–0.24, 0.15]	No

FS_block_	–0.19	0.18	[–0.018, 0.018]	[–0.53, 0.17]	No

IN	–	–	–	–	–

IN_trial_	–0.04	0.14	[–0.014, 0.014]	[–0.31, 0.24]	No

IN_block_	–0.56	0.27	[–0.027, 0.027]	[–1.08, –0.03]	Yes


**Figure 6:**
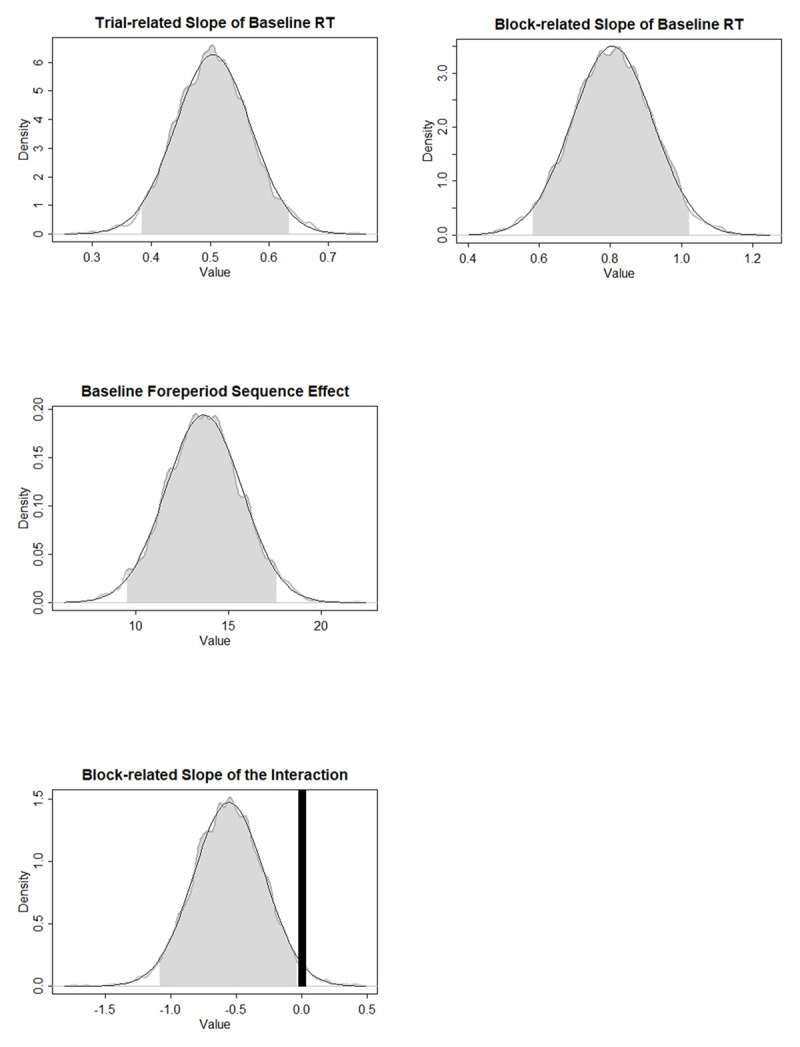
Experiment 3: ROPEs and HDIs of the critical parameters. Gray shades represent the 95% HDIs. Black areas represent the ROPEs to zero. Foreperiod Sequence effect refers to the priming component and the Interaction refers to the arousal component of the sequential foreperiod effect.

**Figure 7:**
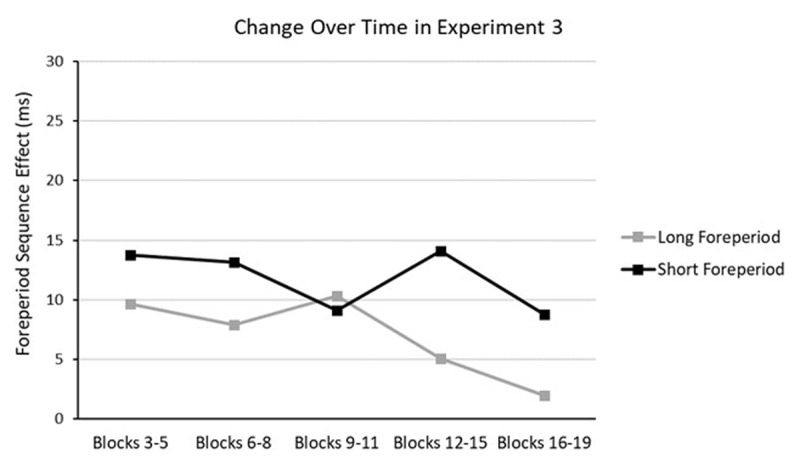
Experiment 3: Foreperiod sequence effect (in ms) as a function of block bin and current foreperiod. Positive values represent shorter RTs with foreperiod repetition.

Figure 6 presents the ROPEs and HDIs of the parameters that were found to be significantly different from zero in Experiment 3. The Current Foreperiod effect was not found to be significantly different from zero. Consistent with Experiments 1 and 2, the short-term priming based SFP effect was different from zero. Although corresponding ROPE and HDI are close to each other, the block-related slope of the arousal-based component was significantly different from zero, which is similar to the result in Experiment 1 but distinct from that in Experiment 2. In terms of the data pattern, it means that the benefit of foreperiod repetition at the longer foreperiod decreases over time across blocks. Figure 7 provides a rough demonstration of this change by plotting the effect of foreperiod sequence at different foreperiods as a function of block bin.

### Discussion

Experiments 1, 2 and 3 provided informative results regarding the SFP effect by using non-aging foreperiod distributions with different pairs of foreperiods. In a long-foreperiod scenario (Experiment 1), both the priming and arousal components were significantly different from zero, with the priming component showing RT benefit of foreperiod repetition and the arousal component reflecting shorter RT when the foreperiod in the preceding trial was shorter. This combination produced an asymmetric SFP pattern, which is more consistent with the predictions of MTP. However, one surprising finding is that the arousal component, which led to a smaller SFP effect at the long foreperiod, was more likely to be emerging later in a block, which is neither predicted by MTP nor the dual-process models. A similar pattern was also found in Experiment 3, in which the arousal component appeared later in the experiment. Compared to Experiments 1 and 3, Experiment 2 had shorter foreperiods. In this case, the SFP effect was dominated by the priming component, supporting the repetition priming account by Capizzi et al. (2015).

Experiments 1, 2 and 3 are also informative with respect to the effect of Current Foreperiod. In Experiment 1, RT at the long foreperiod increased more over time than that at the short foreperiod, which is neither predicted by MTP nor the dual-process models. This finding could be explained by the fatigue effect as shown in Bjørklund (1992). Such long-term change was not found in Experiments 2 and 3, in which the foreperiods involved are shorter than those in Experiment 1. This implies that long foreperiods could be more vulnerable to the influence of fatigue. With regard to the short-term Current Foreperiod effect, the modeling results provide evidence consistent with the conclusion of Han and Proctor (2022b). Thus, further discussion about it is not included in the current paper.

## Experiment 4

The purpose of this experiment was to test whether the findings regarding the SFP effect in a non-aging foreperiod distribution (Experiment 1) could be replicated in an aging distribution. The foreperiods used in this experiment were identical to those in Experiment 1. Based on dual-process models, the SFP effect in an aging foreperiod distribution (e.g., uniform) is modulated by endogenous preparation, which should lead to a different pattern than that with a non-aging distribution. According to the multiple trace theory, changing the foreperiod distribution does not influence the underlying mechanism of the SFP effect. Therefore, there is not supposed to be any fundamental differences between patterns in different foreperiod distributions. Thus, the difference between the modeling results in Experiments 1 and 4 were informative for comparing these two types of temporal preparation models. Also, we expected to find a fatigue effect larger at the long foreperiod, as shown in Experiment 1, because the foreperiods involved were identical.

### Method

#### Participants

Seventy-six students (30 male, 46 female, age range: 18–26 years, mean: 18.88 years, SD: 1.3 years) from the same participant pool participated, none of whom had participated in the three prior Experiments. The reason for using this sample size was to resemble the case in Experiment 1.

#### Apparatus, Stimuli, Procedure

The apparatus, stimuli, procedure and the modeling process of Experiment 4 were the same as those of Experiment 1, except that a different foreperiod distribution (uniform) was used. There was one practice block followed by 15 test blocks. The practice block contained 12 trials, 6 with the shorter foreperiod and 6 with the longer foreperiod to provide a general impression about the mapping and the structure of a block. Each test block contained 24 trials, 12 with the shorter foreperiod and 12 with the longer foreperiod. After vocally introducing the experiment procedure and requirements, the experimenter stayed out of the room to obey the social distancing guidance of the Covid-19 protocol, which was not in effect when Experiment 1 was conducted.

#### Predictions from Existing Models

The multiple trace theory has similar predictions in uniform and non-aging foreperiod distributions. Based on this theory, we expected to find a similar priming-arousal combination as shown in Experiment 1. Dual-process models, on the other hand, predict that the endogenous preparation process should modulate the SFP effect. More specifically, the models predict that this modulation should be based on the relative proportions of different foreperiods. Thus, this modulation is not supposed to be influenced by the number of previous trials and blocks.

### Results

All trials with RT < 100 ms or >1000 ms were regarded as outliers and excluded from the analyses. The first trial of each block, trials following an incorrect response were also discarded. The proportion of excluded regular trials is 9%.

Model comparison results indicate that *Model1* (the full model) has the best performance in predicting future data. The 95% interval of the WAIC difference between *Model1* and the second best one (Model1’) is [3.53, 40.87], supporting the results based on Akaike weights (1 for *Model1* and 0 for the others). Therefore, we used *Model1* for further testing.

**Figure 8:**
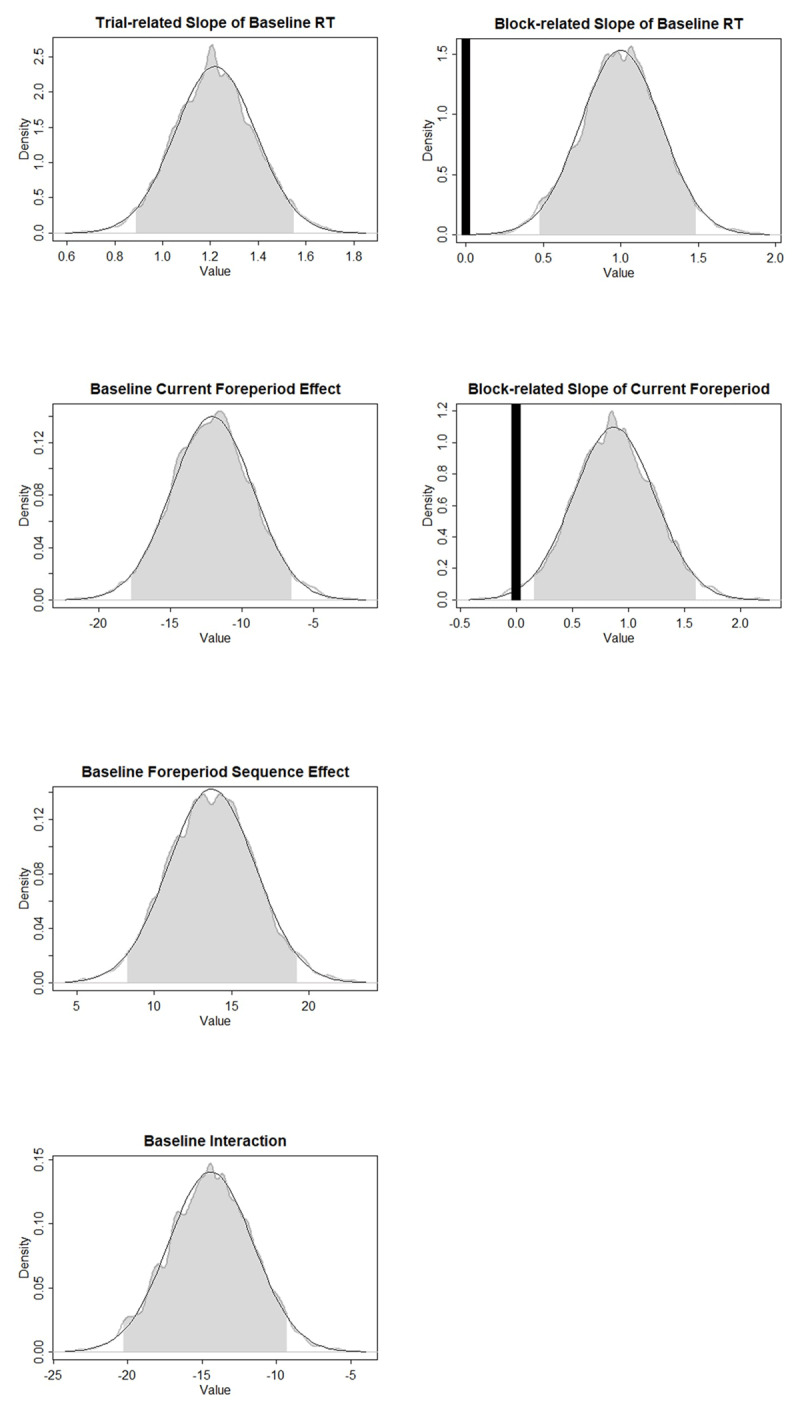
Experiment 4: ROPEs and HDIs of the critical parameters. Gray shades represent the 95% HDIs. Black areas represent the ROPEs to zero. Foreperiod Sequence effect refers to the priming component and the Interaction refers to the arousal component of the sequential foreperiod effect.

Table 4 shows the parameter characteristics of *Model1*, and Figure 8 presents the ROPEs and HDIs of the parameters that were found to be significantly different from zero. A fatigue-like effect on overall RT was shown at both trial and block level. In contrast with Experiment 1, the baseline foreperiod-RT function in Experiment 4 was found to be decreasing. Similar to that in Experiment 1, a block-level long-term modulation on the Current Foreperiod effect was found, indicating a larger RT increase at the longer foreperiod. Posterior distributions show that, combining the short-term and long-term influences, the probability of the foreperiod-RT function being decreasing in the last block is 0.49, which means that the direction of the Current Foreperiod effect in the last block is quite uncertain. Similar to Experiment 1, the priming component of the SFP effect showed a repetition benefit at baseline and did not change over time. Consistent with the model comparison results, the arousal component of the SFP effect was significantly different from zero. Posterior distributions demonstrate that the probability of the baseline SFP effect at 1400 ms favoring foreperiod repetition is 0.44, indicating that the prior foreperiod had little influence when the current foreperiod was long.

**Table 4:** Posterior characteristics of the parameters in Experiment 4. CF refers to the effect of the current foreperiod (positive values indicate that RT was shorter at the short foreperiod). For the sequential foreperiod effect, FS effect refers to the priming component (positive values indicate that RT was shorter with foreperiod repetition), and the IN refers to the arousal component (negative values mean that RT was shorter when the foreperiod of the preceding trial was short).


PARAMETER	MEAN	SD	ROPE TO ZERO	95% HDI	REJECT ZERO?

Baseline_trial_	1.22	0.17	[–0.017, 0.017]	[0.89, 1.56]	Yes

Baseline_block_	1.00	0.26	[–0.026, 0.026]	[0.48, 1.51]	Yes

CF	–12.07	2.85	[–0.285, 0.285]	[–17.72, –6.40]	Yes

CF_trial_	0.04	0.24	[–0.024, 0.024]	[–0.42, 0.50]	No

CF_block_	0.87	0.36	[–0.036, 0.036]	[0.14, 1.59]	Yes

FS	13.71	2.81	[–0.281, 0.281]	[8.33, 19.32]	Yes

FS_trial_	–0.05	0.23	[–0.023, 0.023]	[–0.50, 0.39]	No

FS_block_	0.22	0.35	[–0.035, 0.035]	[–0.47, 0.92]	No

IN	–14.40	2.84	[–0.284, 0.284]	[–20.07, –9.04]	Yes

IN_trial_	–0.14	0.33	[–0.033, 0.033]	[–0.78, 0.51]	No

IN_block_	–0.55	0.49	[–0.049, 0.049]	[–1.50, 0.42]	No


### Discussion

Experiment 4 provided some informative results regarding how foreperiod distribution influences the SFP effect. Consistent with Experiment 1, both the priming and arousal components were significantly different from zero, which produced an asymmetric SFP pattern. Also consistently, in the priming component, only the intercept (short-term part) was significant. However, although the arousal component was significant in both experiments, it was dominated by the short-term part in Experiment 4. In Experiment 1 and 3, in which a non-aging foreperiod distribution was used, the arousal component was driven by the trial- or block-related parameters. The first major difference is also reflected in the model comparison results. In Experiment 4, the full model (Model1) performed better than *Model1’*, which is opposite to the cases in Experiments 1, 2, and 3.

Regarding the foreperiod-RT function, Experiment 4 successfully confirmed the finding from Experiment 1 and the predictions from previous accounts. Based on the results of Experiments 1, 2 and 3, we proposed that the long-term change of the Current Foreperiod effect was caused by fatigue of maintaining temporal preparation at long foreperiods, which should also be observed in a uniform distribution. Such an explanation was confirmed by the results of Experiment 4, which showed the same direction of long-term change as in Experiment 1.

The short-term part of the Current Foreperiod effect was consistent with both MTP and dual-process models. Because in a uniform distribution, there are equal numbers of trials with different foreperiods, this memory-based long-term effect should be dominated by the relative lengths of foreperiods. MTP assumes that previous long-foreperiod memory traces produce inhibition at the short foreperiod, whereas previous short-foreperiod memory traces do not affect the activation at the long foreperiod. Therefore, MTP predicts a decreasing foreperiod-RT function in a uniform distribution. According to dual-process models, in a uniform distribution, the time hazard increases over time, also leading to a decreasing foreperiod-RT function.

## General Discussion

Temporal preparation with a variable foreperiod has been regarded as a combination of both the influence of the current interval between the warning signal and the target stimulus and the impact from previous preparation experiences. In this type of construct, the sequential influence of foreperiod duration is assumed to reflect a short-term product caused by the arousal state inherited from the preceding preparation (Vallesi, 2010) or the memory of the previous foreperiod (Capizzi et al., 2015; Los et al., 2014). This effect has been assumed to not change over time in an experiment, but this assumption has not yet been explicitly tested.

One reason for the lack of direct evidence is that most statistical tests in the literature were on mean RTs across all trials, which would conceal any change of foreperiod effects within an experiment. Some exceptions are studies of Visalli et al. (2019) and Kruijne et al. (2022). The former modeled the update of time hazard information using a Bayesian way of thinking and the latter used a rolling regression method to demonstrate the change of temporal preparation within an experiment. Compared to these two cases, the current study has two major contributions. First, both Visalli et al.’s and Kruijne et al.’s experiments involved the change of scenarios, or more specifically the change of foreperiod distribution whereas the current study focused on the change of foreperiod effects within the same distribution. Second, the main research purpose of Visalli et al. and Kruijne et al. was to investigate the change of foreperiod-RT function. However, we put the most emphasis on the SFP effect, which has been regarded as only influenced by the foreperiod in the preceding trial.

In the current study, we investigated trial-level data, with the aim of detecting any existing trial- and block- level change of not only the foreperiod-RT function but also the SFP effect. The application of Bayesian modeling allowed us to compare models with or without the trial- and block-related parameters. Although we only tested linear relations between variable-foreperiod effects and the number of trials or blocks, the results from model comparison showed that including the trial- and block-related parameters always improved model performance based on the Akaike weights. This improvement should not be attributed simply to adding more parameters because the model comparison we used considers both the model fit to the data and the redundant model flexibility that allows the model to fit noise. A clear example would be the fact that in Experiments 1, 2, and 3, *Model1* was outperformed by *Model1’*, while the latter had one less parameter than the former.

With this Bayesian modeling method, the current study revealed that the SFP effect could be modulated by distinct factors in different foreperiod distributions. With a non-aging distribution, the SFP effect was more consistent with the predictions of the repetition priming account when extremely short foreperiods (Experiment 2) were used. With longer foreperiods (Experiments 1 and 3), the SFP effect was asymmetric, which is more consistent with the prediction of MTP. However, different from the assumption of MTP, the asymmetry emerged later in a trial block (Experiment 1) or later in an experiment (Experiment 3). With regard to the foreperiod-RT function, we found a larger RT increase at the long foreperiod as the numbers of previous trials or blocks increased. This pattern could be found in both aging and non-aging foreperiod distributions and was only significant in a long-foreperiod scenario (Experiments 1 and 4). In the following two sections, implications from these findings are discussed.

### SFP Effect in Different Foreperiod Distributions

The BRAC framework of Frings et al. (2020) proposes that re-encountering one of the features in the event file leads to automatic retrieval of all the elements of the previous episode. The SFP effect of temporal preparation could be a demonstration of this mechanism. It has been shown by Steinborn et al. (2009) that changing the modality of the warning signal made the SFP effect less pronounced. However, because of the various findings in the area of temporal preparation (Capizzi et al., 2015; Vallesi & Shallice, 2007; Langner et al., 2018), the mechanism behind the SFP effect remained unclear.

One of the major debates is whether the SFP effect is modulated by the hazard function in an aging foreperiod distribution. Based on MTP, the SFP effect is simply the result of retrieving the memory trace of the preceding trial, which means the SFP effects in different foreperiod distributions are caused by the same mechanism (Los et al., 2014). According to dual-process models, the hazard function leads to an asymmetric SFP effect in aging foreperiod distributions. When information from the hazard function is unavailable or uninformative, the SFP effect will be symmetric. In Vallesi and Shallice (2007), children at the age of 4 years showed symmetric SFP effect with RT benefit when the foreperiod in the preceding trial was short. In Capizzi et al. (2015), a non-aging foreperiod distribution led to symmetric SFP effect with RT benefit for foreperiod repetitions. In other words, although different dual-process models have consensus about the influence of the hazard function, they have distinct predictions regarding the mechanism behind the symmetric pattern of the SFP effect.

The present study included both arousal- and priming-based components in the SFP effect and tested the significance of these parameters. Except for those in Experiment 2 (50 ms & 200 ms), the modeling results showed support for the predictions of MTP. However, a surprising inconsistency was found between different foreperiod distributions. With a non-aging foreperiod distribution, the short-term SFP effect was dominated by the priming component, with the asymmetry emerging in later trials of a block (Experiment 3) or in later blocks of an experiment (Experiment 1). This was different from the case with a uniform distribution (Experiment 4), in which the asymmetry was evident from the beginning and was not significantly modulated by the number of previous trials or blocks.

This observed inconsistency could have several different explanations. First, it could have been caused by our modeling method. One could argue that the reason for finding a long-term asymmetry in Experiments 1 and 3 was that we used the *Model1’* instead of the full model. It is true that in the *Model1’* of Experiment 4, in which the intercept (short-term parameter) was removed, both the trial- and block- related parameters of the arousal component were significantly different from zero, which could be falsely regarded as evidence supporting a long-term asymmetry in a uniform distribution. However, such a rationale is not able to explain the difference in model comparison results. In Experiments 1 and 3, *Model1’* performed better than the full model, whereas in Experiment 4, the full model had better performance. Moreover, in the full model of Experiment 1 with the intercept of the arousal component included, the trial-related parameter was also marginally significant with an HDI of [–1.16, .06], whereas the same parameter in Experiment 4 had an HDI of [–.78, .51]. Therefore, the choice of model is not adequate to explain the result difference between foreperiod distributions.

If the difference in the source of the asymmetry between foreperiod distributions was not caused by methodological factors, then it should have crucial implications. First, it implies that the SFP effect could have been caused by different mechanisms in different foreperiod distributions, which is more consistent with the assumptions of dual-process models than MTP. In other words, although based on the results of repeat-measures ANOVA in Han and Proctor (2022b), the SFP effect in Experiment 1 still showed an overall significant asymmetry similar to the case in a uniform distribution. They could be distinct phenomena with different time courses of emergence. Such distinction is not possible from the results of analyzing the mean RTs, which demonstrates the advantage of conducting Bayesian modeling on trial-level data.

Second, the long-term asymmetry found in Experiments 1 and 3 also implies a dissociation of factors underlying the SFP effect in non-aging foreperiod distributions. In Experiment 2, in which the foreperiods were both extremely short, such long-term asymmetry was not found while the short-term repetition benefit was still significant. Combining the results from Experiments 1, 2, and 3, this long-term asymmetry could be caused by some mechanism that needs some time between the warning signal and the imperative stimulus to develop. Such a mechanism is different from what caused the repetition benefit, which can even function with extremely short foreperiods. Such a distinction provides some support for the repetition-priming account by Yashar and Lamy (2013) and Capizzi et al. (2015), which argues that the SFP effect in a non-aging foreperiod distribution is dominated by the automatic foreperiod priming effect of the preceding trial. Finally, although there is reason to believe that this long-term asymmetry is not the result of what causes the asymmetry in an aging foreperiod distribution, the underlying mechanism of it remains unclear. Further investigation on this phenomenon is beyond the scope of the current study and could be the purpose of future research.

### Implications From the Observed “Fatigue” Effect

Mattiesing et al. (2017), Los et al. (2017), and Crowe and Kent (2019) found that the foreperiod-RT function in the current block could be modulated by the foreperiod distribution of the preceding block. Such a transfer effect was also found to vanish gradually. In the current study, we found that even within the same foreperiod distribution, the foreperiod-RT function also changed over time to a more increasing or a less decreasing extent, which was consistent across different foreperiod distributions. Moreover, it was shown in the present study that the fatigue effect was evident in relatively long foreperiods (Experiments 1 and 4) but not with shorter ones (Experiments 2 and 3).

The seemingly most likely explanation for this observed phenomenon is fatigue. Some previous studies have used fatigue to explain the extra slowing down of RT at the long foreperiod. However, the evidence is mixed. Bjørklund (1992) used a foreperiod range of 0.5 s to 5 s and a task duration of 80 minutes to investigate the effect of time on task on the foreperiod effect. An extra increment in RT was observed at 5-s foreperiod after 40 minutes of time on task. Langner et al. (2010) used three distinct foreperiods (1 s, 3 s, 5 s) and a task duration up to 51 minutes and observed no interaction between time-on-task and Current Foreperiod. An experiment in the current study took between 35 to 40 minutes. Thus, it is unclear why the fatigue effect was shown in the current study and Bjørklund’s but not in Langner et al. (2010).

The assumption that preparing for a long foreperiod is more effortful and more vulnerable to fatigue effect has some important implications. First, it implies the existence of endogenous preparation, which is based on the hazard function as argued by dual-process models. Vallesi et al. (2014) found that introducing a resource-consuming secondary task modulated the foreperiod-RT function to a less decreasing extent but left the SFP effect unaffected. However, Van Lambalgen and Los (2008) also involved a secondary task but observed the opposite pattern with the SFP effect diminished but the foreperiod-RT function remaining the same. Therefore, whether the effect of Current Foreperiod is caused by a resource-consuming mechanism (e.g., endogenous preparation) remains unclear. Moreover, based on dual-process models, endogenous preparation should be discouraged in non-aging foreperiod distribution. However, the long-term effect on foreperiod-RT function was observed in Experiment 1, which means that this effect might not be related to the change in foreperiod distribution.

Another possible explanation for this effect is that preparation for a long foreperiod itself is effortful and is more vulnerable to the effect of fatigue. This is in line with the arousal-based model by Vallesi (2010), which assumes that more energy or effort is needed to maintaining arousal for a longer period of time. This explanation can also be related to the finding of a long-term arousal component by assuming that as fatigue accumulates, the difference in energy or effort in maintaining arousal for different foreperiods could be increased as the preparing for a long foreperiod becomes even more effortful and that for a short foreperiod remains unaffected. This would lead to an increased modulation from arousal. However, such connection between these two phenomena was not found in Experiment 3, where the long-term arousal component was significant, but the fatigue effect was not. Therefore, more evidence is needed to conclude about the essence of this change of foreperiod-RT function over time within an experiment.

Finally, it is worth noting that the current study is not immune to some limitations. First, all the short-term effects were represented as the intercept of a linear function. The precision of these estimates is affected by how well the function resembles the actual tendency of the data. A linear function could be an oversimplification of the actual tendency, which could have led to errors in estimating the values of the intercepts. Thus, future studies should explore other functions so that the errors can be decreased by using a better model to represent the data. Second, the modeling process did not include the data from the practice blocks (Experiments 1, 2 and 4), which could have added error in estimating the baseline or short-term effects. An alternative design could use tasks without the warning signal for practice trials as an attempt to eliminate the influence of this extra exposure. Although faced with such limitations, the findings of the study are still valuable and meaningful in that they point out the existence of the long-term patterns in a quantitative manner. Also, the use of Bayesian modeling provides an example of obtaining extra information from behavioral data, which should be emphasized more in future empirical research.

## Conclusion

The current study used Bayesian modeling on trial-level data from several variable foreperiod experiments and found that the cause of the SFP effect could be distinct between short- and long-foreperiod scenarios and between different foreperiod distributions. Our findings indicate that sequential modulation, which is attributed to feature binding and retrieval by the BRAC framework, could have different underlying mechanisms depending on the task scenario.

## Data Accessibility Statement

The trial-level data of the four experiments and the codes for the modeling process in the current study are available on OSF (Open Science Framework) through the following link: https://osf.io/um34f/?view_only=a3a7d05f2798445885eb2b88a795640f.

## Footnotes

This step was taken because the short-term component of β_SFP_arousal_ was found to be nonsignificant in Experiments 1, 2 and 3. The model comparison results suggest that excluding this component improved model performance.
